# Primary Hyperparathyroidism: To Evaluate Benefit of Ultrasound and Tc99m-SESTAMIBI Scan in Localizing Abnormal Parathyroid Gland Before Surgery, in a Secondary Care Hospital

**DOI:** 10.7759/cureus.10155

**Published:** 2020-08-31

**Authors:** Habib U Rehman, SenthilKumar Krishnasamy, Jamal Rabbi, Mamoon Qadir, Yasmeen Rafique, Fahd Mian, Quratulain Yousuf

**Affiliations:** 1 General Medicine, Endocrinology, Kulsum International Hospital, Islamabad, PAK; 2 Endocrinology, Diabetes and Metabolism, Walsall Manor Hospital, Walsall, GBR; 3 Cardiac Surgery, Kulsum International Hospital, Islamabad, PAK; 4 Cardiology, Polyclinic Hospital, Islamabad, PAK; 5 Internal Medicine, Benazir Bhutto Hospital, Rawalpindi, PAK

**Keywords:** primary hyperparathyroidism, tc99m sestamibi scan, ultrasound parathyroid

## Abstract

Introduction

Primary hyperparathyroidism is a common endocrine condition requiring parathyroidectomy for curative management. Localization of parathyroid gland by ultrasound and Tc99m-SESTAMIBI is important to opt for less invasive and comparatively lower complication risk surgery minimal invasive parathyroidectomy (MIP) instead of four-gland exploration surgery.

Aim

To evaluate ultrasound and Tc99m-SESTAMIBI in localization of abnormal parathyroid gland before surgery.

Method and materials

All patients of primary hyperparathyroidism (PHPT) that presented to a secondary care hospital (endocrinology department) from 2015-2019 were recruited retrospectively from electronic fusion system of hospital. Results of ultrasound parathyroid and Tc99m-SESTAMIBI done for localization of abnormal parathyroid gland were analyzed.

Results

Total PHPT patients recruited were 59, mean age 64.2 years, male 11 (18.64%) and female 48 (81.3%). Ultrasound parathyroid was done in 44 patients, Tc 99m-SESTAMIBI was done in 31, both tests were done in 31 patients. Combined concordant adenoma in both tests was seen in 11 (35%) cases which can opt for minimal invasive parathyroidectomy (MIP) with confidence whereas 65% of cases would require either four-gland exploration or further testing like single-photon emission computed tomography-computed tomography (SPECT-CT) or intraoperative parathyroid hormone measurement to opt for MIP.

Conclusion

Combined ultrasound parathyroid and Tc 99m-SESTAMIBI scan was useful in localization of parathyroid adenoma in 11 (35%) patients that can opt for MIP which is a lower complication risk surgery whereas 20 (65%) patients would need further investigation with SPECT-CT or intraoperative parathyroid hormone measurement or four-gland exploration surgery.

Recommendation

Third modality of investigation such as SPECT-CT or intraoperative parathyroid hormone measurement needs evaluation so that more patients can benefit from MIP instead of four-gland exploration surgery.

## Introduction

Primary hyperparathyroidism (PHPT) is a common endocrine disease present in one in 500 of the general population [1]. It is more common in female population and incidence increases with age [2]. It is characterized by excessive secretion of parathyroid hormone resulting in increased serum calcium (>2.6 mmol/l) [3]. Mostly it is due to a benign adenoma (80%), sometimes due to hyperplasia (10-15%) and rarely due to parathyroid cancer (1%) [4]. Most often patients are asymptomatic and are found to have raised calcium on routine blood test but sometimes patients present with complications such as nephrolithiasis, fragility fractures due to osteoporosis or renal failure among others [5,6].

Definitive management of PHPT is by parathyroidectomy which is curative [7]. Localization of abnormal gland is important before surgery which is done by ultrasound of parathyroid gland and Tc 99m-SESTAMIBI scan. Tc 99m-SESTAMIBI scan has sensitivity of 79.1% and specificity of 86.7% in PHPT whereas ultrasound parathyroid has sensitivity of 74% and specificity of 96% [8]. If able to localize abnormal parathyroid gland then minimal invasive parathyroidectomy (MIP) is done which has advantage of minor cervical exploration, shorter hospital stay and small scars as compared to four-gland exploration which is done in cases with failed localization [7].

Aim of the study is to evaluate ultrasound and Tc99m-SESTAMIBI scan in localization of abnormal parathyroid gland in PHPT before surgical intervention.

## Materials and methods

Descriptive cross-sectional study

All patients of PHPT that presented to endocrine department of Walsall Manor hospital NHS trust (secondary care hospital) from 2015 to 2019 were studied retrospectively.

Data was obtained from fusion electronic system of hospital which has access to all inpatient and outpatient labs, imaging and clinic letters. All patients that had ultrasound parathyroid and Tc 99m SESTAMIBI scans done for localization of abnormal parathyroid before surgery from 2015-2019 were obtained and through clinical letters on fusion system PHPT patients were recruited from them. Patients data including demographic profile, ultrasound parathyroid results, Tc 99m SESTAMIBI scan results was entered in Microsoft office excel sheet for analysis (Office 2010).

Inclusion criteria

· Confirmed cases of PHPT seen by endocrinology team in Walsall Manor Hospital.

· Age 18-100

Exclusion criteria

· All patients of secondary hyperparathyroidism and tertiary hyperparathyroidism.

## Results

**Figure 1:**
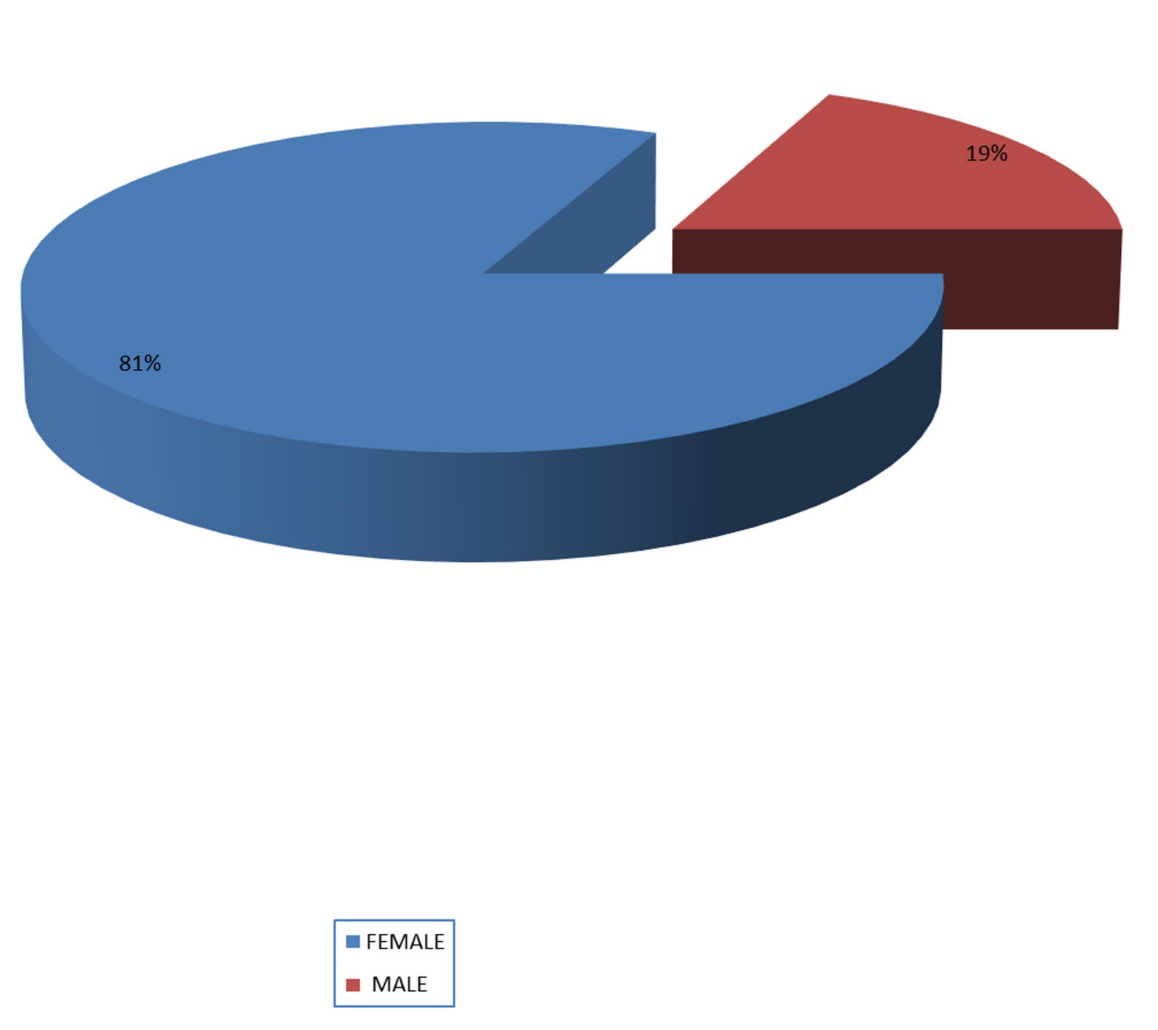
Gender

**Figure 2:**
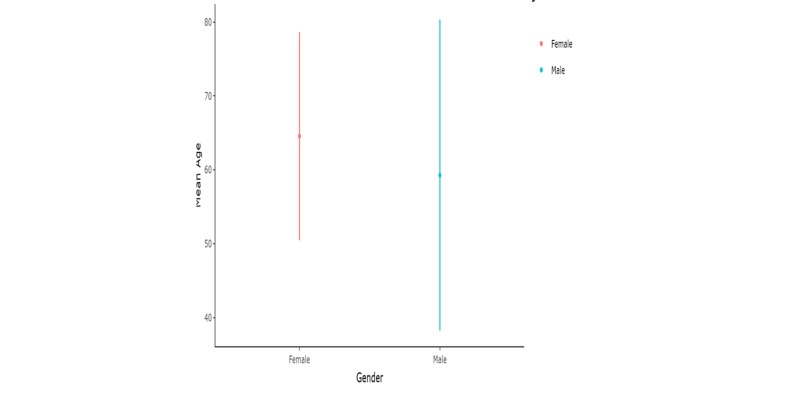
Mean age by gender

**Figure 3:**
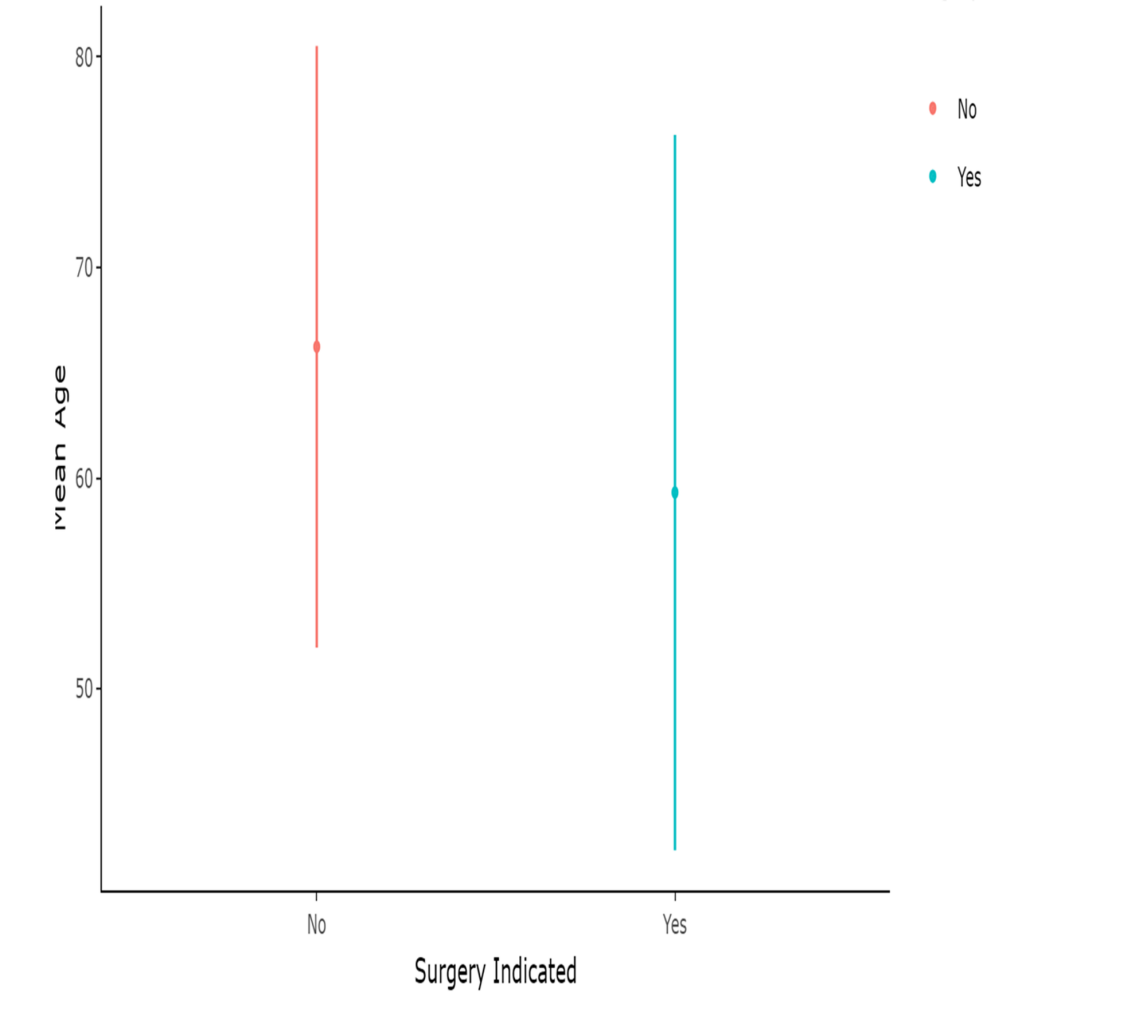
Mean age by surgery indication

Total patients of PHPT seen by endocrinology department in five years were 59, mean age 64.2 years, male 11 (18.64%) and female 48 (81.3%) (Figure 1). Mean age did not differ among males and females (p-value = 0.44) (Figure 2). Mean age also did not differ in patients who had indication of surgery (p-value = 0.13) (Figure 3).

For localization of abnormal parathyroid 44 patients had ultrasound and 31 patients had Tc 99m SESTAMIBI scan done. Among these patients both tests were done in 31 patients.

In patients who had ultrasound parathyroid done, 19 (43.2%) showed adenoma whereas 25 (56.8%) were normal and no hyperplasia or carcinoma was seen. Adenoma of right inferior gland was seen in 11 (25%), left inferior gland in four (9%), left superior in two (4.5%) and right superior gland in two (4.5%) (Table 1).

**Table 1:** Ultrasound and SESTAMIBI scan results

	Ultrasound Parathyroid	SESTAMIBI Scan
Total	44	31
Adenoma	19 (43.2%)	16 (51.6%)
Normal	25 (56.8%)	15 (48.4%)
Hyperplasia	0	0
Parathyroid Cancer	0	0

In patients who had Tc 99m SESTAMIBI scan done, adenoma was seen in 16 (51.6%) whereas 15 (48.4%) were normal. Left inferior gland adenoma was seen in eight (25.8%), right inferior adenoma in seven (22.5), right superior gland adenoma in one (3.2%) and no left superior gland adenoma was seen (Table 1).

Patients who had both ultrasound and Tc 99m SESTAMIBI scan among all the patients were 31. When both tests were done, 20 (64.5%) patients showed adenoma in one of the tests and was concordantly seen in both tests in 11 (35%) whereas both were normal in 11 (35%) (Table 2).

**Table 2:** Combined ultrasound and Tc99m-SESTAMIBI results

	When ultrasound and SESTAMIBI combined
Total patients that had both tests	31
Adenoma seen in either one or both tests (Discordant)	20 (64.5%)
Concordant adenoma seen in both	11 (35%)
Both tests normal	11 (35%)

## Discussion

Primary hyperparathyroidism (PHPT) is a common endocrine condition for which definitive and curative treatment is parathyroidectomy. Surgery is indicated if calcium >2.85 mmol/l, evidence of end organ damage such as renal failure, nephrolithiasis, osteoporosis or if patient has symptomatic hypercalcemia. PHPT is also considered for surgery if opted by patient irrespective of calcium level or organ damage [9]. Surgery is either minimal invasive parathyroidectomy (MIP) or four-gland exploration.

MIP is preferred over four-gland exploration surgery as neck dissection is less, scar is small, less operative time, shorter duration of stay, low risk of recurrent laryngeal nerve injury compared to four-gland exploration [10,11]. For MIP abnormal parathyroid gland localization before surgery is important for which two investigative modalities are used commonly i.e. ultrasound parathyroid and Tc 99m SESTAMIBI scan. Combined sensitivity of ultrasound and Tc 99m SESTAMIBI scan is superior than either of them alone.

Cases in which results are concordant such as both modalities localizing abnormal parathyroid gland to one of the four glands, MIP is opted but if the tests are discordant or normal then four-gland exploration is proceeded with unless other investigative modalities such as single-photon emission computed tomography-computed tomography (SPECT-CT) or intraoperative parathyroid hormone levels are used if facilities are available to do so in specialized centres [11].

In our study, PHPT patients which had both parathyroid ultrasound and Tc 99m SESTAMIBI concordant adenomas were seen in 11 (35%) of 31 patients whereas in 11 (35%) patients both tests were normal and 10 (32%) patients showed adenoma in one of the tests. Based on these only 35% of patients can proceed to MIP with confidence, whereas 65% of patients would require four-gland exploration or other investigative modalities such as intraoperative parathyroid hormone levels or SPECT-CT to localize for MIP.

## Conclusions

In PHPT patients combined ultrasound and Tc 99m SESTAMIBI scans were useful to localize abnormal parathyroid gland for MIP in 35% of the cases whereas 65% of cases needed four-gland exploration surgery to remove abnormal parathyroid gland or further investigative modalities such as SPECT-CT of neck or intraoperative parathyroid hormone measurement which are done in only specialized centers to opt for low-risk surgery MIP.
